# Interpreting biomonitoring data: Introducing the international human biomonitoring (i-HBM) working group’s health-based guidance value (HB2GV) dashboard

**DOI:** 10.1016/j.ijheh.2022.114046

**Published:** 2022-11-07

**Authors:** Shoji F. Nakayama, Annie St-Amand, Tyler Pollock, Petra Apel, Yu Ait Bamai, Dana Boyd Barr, Jos Bessems, Antonia M. Calafat, Argelia Castaño, Adrian Covaci, Radu Corneliu Duca, Sarah Faure, Karen S. Galea, Sean Hays, Nancy B. Hopf, Yuki Ito, Maryam Zare Jeddi, Marike Kolossa-Gehring, Eva Kumar, Judy S. LaKind, Marta Esteban López, Henriqueta Louro, Kristin Macey, Konstantinos C. Makris, Lisa Melnyk, Aline Murawski, Josh Naiman, Julianne Nassif, Nolwenn Noisel, Devika Poddalgoda, Lesliam Quirós-Alcalá, Ata Rafiee, Loïc Rambaud, Maria João Silva, Jun Ueyama, Marc-Andre Verner, Maisarah Nasution Waras, Kate Werry

**Affiliations:** a Exposure Dynamics Research Section, Health and Environmental Risk Division, National Institute for Environmental Studies, 16-2 Onogawa, Tsukuba, Ibaraki, 305-8506, Japan; b Healthy Environments and Consumer Safety Branch, Health Canada, 269 Laurier Ave W, A/L 4908D, Ottawa, ON, K1A 0K9, Canada; c German Environment Agency, Berlin/ Dessau-Roßlau, Wörlitzer Platz 1, 06844, Dessau-Roßlau, Germany; d Center for Environmental and Health Sciences, Hokkaido University, Kita12, Nishi 7, Kita-ku, Sapporo, Japan; e Gangarosa Department of Environmental Health, Rollins School of Public Health, Emory University, 1518 Clifton Road NE, Atlanta, GA, 30322, USA; f VITO NV, Boeretang 200, 2400, Mol, Belgium; g National Center for Environmental Health, Centers for Disease Control and Prevention, Atlanta, USA; h National Center for Environmental Health, Instituto de Salud Carlos III, 28220, Majadahonda, Madrid, Spain; i Toxicological Center, University of Antwerp, Universiteitsplein 1, 2610, Wilrijk, Belgium; j Unit Environmental Hygiene and Human Biological Monitoring, Department of Health Protection, Laboratoire national de santé, 1, Rue Louis Rech, L-3555, Dudelange, Luxembourg; k Institute of Occupational Medicine (IOM), Research Avenue North, Riccarton, Edinburgh, EH14 4AP, UK; m Summit Toxicology LLP, 615 Nikles Dr., Unit 102, Bozeman, MT, 59715, USA; n Center for Primary Care and Public Health, Route de la Corniche 2, 1066, Epalinges-Lausanne, Switzerland; o Department of Occupational and Environmental Health, Nagoya City University Graduate School of Medical Sciences, 1 Kawasumi, Mizuho-cho, Mizuho-ku, Nagoya, 467-8601, Japan; p National Institute for Public Health and the Environment (RIVM), Antonie van Leeuwenhoeklaan 9, 3721 MA, Bilthoven, the Netherlands; q Department of Health Security, Finnish Institute for Health and Welfare, Neulaniementie 4, FI-70210, Kuopio, Finland; r LaKind Associates, LLC, 106 Oakdale Avenue, Catonsville, MD, 21228, USA; s Department of Epidemiology and Public Health, University of Maryland School of Medicine, 655 W. Baltimore Street, Baltimore, MD, 21201, USA; t Department of Human Genetics, National Institute of Health Doutor Ricardo Jorge (INSA), Av. Padre Cruz 1649-016 Lisbon, and Center for Toxicogenomics and Human Health (ToxOmics), NOVA Medical School-FCM, UNL, Rua Câmara Pestana, 6 Ed. CEDOC II, 1150-082, Lisbon, Portugal; u Healthy Environments and Consumer Safety Branch, Health Canada, 269 Laurier Ave W, Ottawa, ON, K1A 0K9, Canada; v Cyprus International Institute for Environmental and Public Health, School of Health Sciences, Cyprus University of Technology, Irinis 95, 3041, Limassol, Cyprus; w U.S. Environmental Protection Agency, Office of Research and Development/Center for Public Health and Environmental Assessment, 26 West Martin Luther King Drive, Cincinnati, OH, 45268, USA; x LaKind Associates, LLC, 504 S 44th St, Philadelphia, PA, 19104, USA; y Association of Public Health Laboratories 8515 Georgia Avenue, Suite 700, Silver Spring, MD, 20910, USA; z Department of Occupational and Environmental Health, School of Public Health, Université de Montréal, C.P. 6128, Succursale Centre-Ville, Montreal, Quebec, H3C 3J7, Canada; aa Department of Environmental Health & Engineering, Johns Hopkins Bloomberg School of Public Health, 615 N. Wolfe Street, Baltimore, MD, 21205, USA; ab Department of Medicine, University of Alberta, 173B Heritage Medical Research Centre, 11207 - 87 Ave NW, Edmonton, AB, T6G 2S2, Canada; ac Occupational and Environmental Health Division, Santé publique France, 12 rue du Val d’Osne, 94415, Saint-Maurice, France; ad Human Genetics Department, National Institute of Health Doutor Ricardo Jorge, Avenida Padre Cruz, 1649-016, Lisboa, Portugal; ae Department of Biomolecular Sciences, Field of Omics Health Sciences, Nagoya University Graduate School of Medicine, Nagoya, 461-8673, Japan; af Toxicology Department, Advanced Medical and Dental Institute, Universiti Sains Malaysia, 13200 Kepala Batas, P. Pinang, Malaysia

**Keywords:** Biomonitoring, Biomonitoring equivalents, Guidance values, Reference values, International society of exposure science

## Abstract

Human biomonitoring (HBM) data measured in specific contexts or populations provide information for comparing population exposures. There are numerous health-based biomonitoring guidance values, but to locate these values, interested parties need to seek them out individually from publications, governmental reports, websites and other sources. Until now, there has been no central, international repository for this information. Thus, a tool is needed to help researchers, public health professionals, risk assessors, and regulatory decision makers to quickly locate relevant values on numerous environmental chemicals. A free, on-line repository for international health-based guidance values to facilitate the interpretation of HBM data is now available. The repository is referred to as the “Human Biomonitoring Health-Based Guidance Value (HB2GV) Dashboard”. The Dashboard represents the efforts of the International Human Biomonitoring Working Group (i-HBM), affiliated with the International Society of Exposure Science. The i-HBM’s mission is to promote the use of population-level HBM data to inform public health decision-making by developing harmonized resources to facilitate the interpretation of HBM data in a health-based context. This paper describes the methods used to compile the human biomonitoring health-based guidance values, how the values can be accessed and used, and caveats with using the Dashboard for interpreting HBM data. To our knowledge, the HB2GV Dashboard is the first open-access, curated database of HBM guidance values developed for use in interpreting HBM data. This new resource can assist global HBM data users such as risk assessors, risk managers and biomonitoring programs with a readily available compilation of guidance values.

## 1. Introduction

Scientists use numerous approaches for evaluating human exposures to environmental chemicals. One approach – human biomonitoring (HBM), or the measurement of chemical biomarkers in human matrices such as urine or blood – has been referred to as the gold standard of exposure characterization (Sexton et al., 2004). Since the 1990s, the use of biomonitoring to characterize human exposures has experienced unprecedented growth (Angerer et al., 2007; Calafat 2016; Paustenbach and Galbraith 2006; Pirkle et al., 1995; Sobus et al., 2015; US EPA 2012), to some degree supplanting other approaches. Biomonitoring has been used in exposure and epidemiology research globally (NRC 2006). It has also been used to obtain nationally representative information on human exposures to a large number (e.g., hundreds) of chemicals (Apel et al., 2017; Bastiaensen et al., 2021; Centers for Disease Control and Prevention, 2022; Fillol et al., 2021; Gilles et al., 2021; Health Canada 2021; Hong et al., 2021; Jeon et al., 2021; Jung et al., 2022; Liao et al., 2021; Schoeters et al., 2017; Schulz et al., 2021; Seifert et al., 2000; Seo et al., 2021; VITO 2022).

HBM data can provide insight into overall population exposures (Angerer et al., 2007, 2011). HBM data or biomarker concentrations from a sample population may also be compared to reference concentrations from national surveys or large population studies. HBM data from such surveys or studies constitute a relevant source to determine reference values (RVs). RVs are particularly relevant in the absence of HBM health-based guidance values (HB2GV), either when such values are not available or cannot be derived (Vogel et al., 2019). The RVs are based on the upper end of the exposure distribution, such as 95th percentile concentrations and may be used for comparisons (Vogel et al., 2019).

Risk assessors can also compare biomarker concentrations measured in specific contexts or populations to health-based guidance values derived from human or animal data using uncertainty factors or modelling approaches, such as physiologically-based pharmacokinetic (PBPK) modelling. Most guidance values (with a few exceptions described later in this paper) are derived from toxicological studies that use dose information (e.g., mg/kg bw/day of a chemical entering the body), but biomonitoring data describe the bioavailable concentration from all sources in a given human matrix (e.g., ng/mL blood). Connecting a dose such as a Reference Dose (RfD) to a biomonitoring concentration requires additional processes. Specifically, modelling efforts can convert dose-based guidance values to concentrations in blood or urine (Hays et al., 2008; Pletz et al., 2020). These efforts have resulted in a large number of human biomonitoring health-based guidance values (HB2GVs), but to locate these values, interested parties need to seek them out individually from publications, governmental reports, websites and other sources. Until now, there has been no central, international repository for this information. Thus, a tool is needed to help researchers, public health professionals, risk assessors, and regulatory decision makers quickly locate relevant data on numerous environmental chemicals.

An effort to develop a repository for health-based guidance values to facilitate the interpretation of HBM data started with the formation of the International Biomonitoring Network (IBN) in 2018. The IBN objectives were to enable knowledge exchange, collaboration, and harmonization across international biomonitoring programs (St-Amand, 2021; Nassif and St-Amand 2021). In 2020, the International Human Biomonitoring Working Group (i-HBM), proposed by the IBN, became formally affiliated with the International Society of Exposure Science (ISES).

The i-HBM’s mission is to promote the use of population-level HBM data to inform public health decision-making by developing harmonized resources to facilitate the interpretation of HBM data in a health-based context (https://intlexposurescience.org/i-hbm/).

A first step was the development of a free, on-line repository (the “Human Biomonitoring Health-Based Guidance Value (HB2GV) Dashboard”) for health-based biomonitoring guidance values. The objectives of the Dashboard are:

- To be an open-access, curated database of biomonitoring guidance values developed for use in interpreting and understanding human biomonitoring data for the general population;
- To provide a user-friendly search tool for human biomonitoring guidance values for specific chemicals and/or biomarkers of exposure; and
- To assist users (risk assessors, risk managers and biomonitoring programs) with the interpretation of HBM data by allowing users to compare population-level data to the guidance values and providing standardized outputs in the form of figures and descriptive text.

This paper provides a description of the HB2GV Dashboard including the methods used to compile the values contained in the database, and how the Dashboard can be accessed and used. Finally, we offer thoughts on caveats with using the Dashboard for interpreting human biomonitoring data.

## 2. Methods

To develop the database, we conducted literature searches during two periods: during March 2020 to October 2020, and February 2022 to March 2022 using PubMed and Google Scholar. Although these literature searches were non-systematic, we targeted several search terms that sought to capture as many health-based biomonitoring guidance values as possible, including “biomonitoring”, “biological monitoring”, “blood guidance values”, “guidance values”, “biomonitoring equivalent(s)”, “human biomonitoring values”, “HBM”, “HBM-I”, “HBM-II”, and “HBM-GV”. Of note, we did not target search terms for occupational exposures or for nutritional or medicinal chemicals. In addition to literature searches, we also consulted websites of certain organizations, including Health Canada and the HBM Commission of the German Federal Environment Agency (https://www.umweltbundesamt.de/en/topics/health/commissions-working-groups/human-biomonitoring-commission-hbm-commission), the Human Biomonitoring for Europe (HBM4EU) initiative (https://www.hbm4eu.eu/), and i-HBM Working Group member affiliations.

We extracted information from the identified literature to populate the Dashboard. While the preponderance of the Dashboard data (e.g., biomarker name, biological matrix, biomonitoring guidance value) were taken directly from the associated primary publications, some information required coding or re-coding for the purposes of consistency (e. g., risk level associated with guidance values developed from cancer endpoints).

The Dashboard includes numerous commonly used terms for HBM guidance values; the terminology and acronyms are described in (Table 1).

The HB2GV Dashboard was developed in R (version 4.1.2, R Foundation for Statistical Computing, Vienna, Austria) using the Shiny Applications package (version 1.7.1, RStudio Inc, Boston, MA, USA). The database will be updated on an annual basis as new guidance values are identified.

## 3. Results

Five hundred eighty eight (588) biomonitoring guidance values were identified from 58 sources.

The Dashboard was designed with a landing page (https://intlexposurescience.org/i-hbm/) that features the database of guidance values shown in a table, as well as a sidebar with multiple reactive search filter options that can be selected by the end user.

The Dashboard has two main functionalities. The first is a Guidance Value search function. The user can search for chemicals by chemical group, chemical name or CAS RN, biomarker name, matrix (e.g., urine, serum), or the units of measure. As the user selects search filters, the table automatically updates with only the relevant guidance values. The following information is shown in the table: Chemical, Chemical CAS RN, Biomarker, Matrix, Group, Exposure Guidance Value Info (Type, Value, Risk Type, Source) and Biomonitoring Guidance Value Info (Type, Value, Units, Reference). Either the complete or the filtered database can be downloaded in a spreadsheet (XLSX) format. When the user selects one chemical, biomarker, matrix, and units from the filters, a figure will be automatically generated below the table that depicts the applicable guidance values as horizontal lines at the appropriate concentrations.

The second main functionality is a comparison feature. The user can input one or more biomonitoring-based chemical concentration(s) of their choosing and compare it with the available guidance values. The user can provide custom labels that describe those concentrations, such as the collection period, name of the biomonitoring program, or citation for the biomonitoring study. These results and labels will be added as bars to the figure in the Dashboard, which allows the user to visually compare them against the applicable guidance values. In cases where chemical concentrations representative of the Canadian population are available, users can import results from the Canadian Health Measures Survey (CHMS) directly into the figure for comparison purposes. The figure, with or without accompany comparative biomonitoring concentrations, can be downloaded as an image (PNG) file. A user guide containing more detailed instructions and the abbreviations list are also made available on the Dashboard. The Dashboard is hosted online through RStudio’s shinyapps.io platform.

## 4. Discussion

The HB2GV Dashboard aggregates existing guidance values and facilitates understanding of how they may be used as screening tools in the interpretation of HBM data. The Dashboard is for informational purposes only and is not intended for drawing conclusions regarding health risk for individuals. For example, it would be appropriate to employ population data in the Dashboard to inform chemical prioritization for further follow up.

It should be noted that the guidance values have been developed by researchers from different sectors (private, government) and areas of expertise (toxicologists, PBPK modelers), and with different funding sources (private and public) and different levels of confidence underpinning the values. These specific details have not been incorporated into the Dashboard database. It is therefore incumbent upon the Dashboard user to examine the chemical-specific publications cited in the Dashboard to determine whether the guidance values selected are fit for the user’s purpose. For this reason, a direct link to the source of each HB2GV is provided and can be accessed from the Dashboard (or in the downloaded XLSX file).

Of note, researchers and government agencies often interpret biomonitoring data using RVs. While understanding how chemical levels in a sample population or individual relate to population levels can be useful for a variety of purposes (e.g., better understanding of possible sources of exposure or populations that warrant additional study or outreach (Vogel et al., 2019)), comparisons to exposure RVs do not offer health-related information. It is critical that this distinction be understood and recognized when using guidance values to interpret HBM data.

The Dashboard will be updated as new guidance values become available. It is our hope that this repository will facilitate already existing and innovative screening approaches that orient prioritization of future efforts (Aylward et al., 2013; Faure et al., 2020; St-Amand et al., 2014). A link is provided in the User Guide for individuals to provide information and documentation on new or otherwise missing guidance values so that they can be added to the Dashboard.

### 4.1. Confidence in HB2GVs

The HB2GVs are each unique in terms of the toxicological and epidemiological data used for their derivation, as well as the models used to develop them; not all of these data and models are of equal quality. Thus, confidence in the derived HB2GVs will vary from one value to the next. Assessing confidence in a given value is a critical step in terms of its use. Yet the process of assigning confidence in these values is fraught with difficulty and is very much reliant on expert judgment. As done with previous efforts to assess confidence in various types of data (LaKind et al., 2014; International Programme on Chemical Safety, 2008) and RVs (US EPA, 1993, 2005), it would be recommended that confidence in HB2GVs be categorized as high, medium, or low (Apel et al., 2020; Hays et al., 2008). However, it is important to note that the processes for assessing high, medium, or low confidence are similar, but not identical for BEs, HBM-I, HBM-II, and HBM-GV values (Hays et al., 2008; Apel et al., 2017, 2020); guidance for the HBM-I and HBM-II values is much more explicit. Further, not all HB2GVs have been assigned a confidence level.

Therefore, the current version of the Dashboard does not include the existing available confidence assessments. Future iterations of the Dashboard may include confidence categories. Several factors that will need to be considered have been described (LaKind et al., 2014) and include study design aspects such as analytical considerations, and sample collection and handling issues. At this time, it is incumbent on the user to examine the underlying literature that describes the development of the HB2GVs in order to assess the confidence in the value, and to put risk estimates in the context of underlying uncertainty.

### 4.2. Interpretation of HB2GVs

Availability of health-based guidance values, as well as RVs, are useful for the interpretation, risk assessment, and comparison of any biomonitoring data. However, it is important to note that comparing HBM data to nationally representative RVs or health-based guidance values can present major interpretive challenges. Issues include, but are not limited to:

- Biomonitoring results provide information on integrated pathways and routes of exposures (Albertini et al., 2006). Thus, HBM data do not provide information on *individual* pathways or routes of exposure. This presents difficulties for those seeking to identify and limit important avenues for human exposures. In this case, the evaluation of information obtained by means of questionnaires within the framework of a survey can be helpful.
- Chemical concentrations in human matrices are inextricably tied to the physiological half-life of the chemical, the time between the last exposure and sampling of matrix, and the nature of the exposure (e. g., constant versus episodic). For many chemicals, half-lives are short (on the order of hours) and data on time since last exposure are often lacking (Teeguarden et al., 2011). This can make use of the HBM data for interpreting human exposure difficult. For example when a chemical concentration is non-detectable, it could imply no exposure or alternatively it could mean that the sample was collected after the chemical was already excreted.
- Many studies include a single (spot) blood or urine sample, generally due to cost and participant burden considerations. A single sample often will not represent long-term exposures and can result in exposure misclassification, especially for chemicals with short physiological half-lives and infrequent exposure patterns (LaKind et al., 2019; Pleil et al., 2013; Verner et al., 2020).
- Placing HBM data into context by comparing results with datasets such as those from national surveys provides information on relative exposures and if the sample size of a study is large enough, the HBM data can represent the population’s exposure status but does *not* provide information related to health.
- HBM data should be derived from laboratories with extensive QA/QC programs that successfully participate in established laboratory harmonization schema (e.g., “round robin” studies, data harmonization programs, external proficiency testing, quality assessment programs).
- Serum concentrations of many persistent halogenated organic pollutants are reported both lipid-corrected and on wet-weight basis (not lipid-corrected) (Bernert et al., 2007). Similarly, urinary concentrations are often corrected for urine dilution with creatinine or specific gravity. Dashboard users will need to ensure that the comparison between guidance values and their biomonitoring data are reported using a common metric (uncorrected or corrected) (Barr et al., 2005). It is for this reason that users *must* select only one unit of measure before generating a figure and making a comparison in the Dashboard.

The HBM values in the Dashboard – with the exception of the blood lead reference value (CDC 2021) – are not meant to be used for comparison with an individual’s biomonitoring data. This is especially true when only a single sample for an individual has been obtained. Many factors influence a single measure and may make it unrepresentative for estimations of overall exposure. These factors include (ACGIH 2001; Aylward et al., 2014; LaKind et al., 2008): physiological makeup and health status, exposure elements such as co-exposures and routes of exposures, and methodological issues (e.g., specimen contamination or degradation during collection and storage). Further, the Reference Doses (RfD) that form the foundation for estimation of GVs are not designed to evaluate individual risk, but rather are meant to assess population risk. The HBM-I and HBM-II values are in Germany also used for individual health counseling, addressing uncertainties and limitations.

Interestingly, there are substances with multiple Dashboard Values. This is the case for a well-studied plasticizer, di-(2-ethylhexyl) phthalate (DEHP), which has 36 entries. To properly interpret the Dashboard in these cases one must have a clear understanding of differences in the various GVs available, how GVs were derived, and their intended use and application. On the other hand, the Dashboard can also reveal data gaps where chemicals of interest have no health-based guidance values available. This can help prioritize future efforts and target resources in deriving new values in the future.

The Dashboard user should also be aware of the possibility that guidance values may be updated from time to time. Specifically, existing guidance values should be checked periodically to ensure that they are up to date. For example, for so called legacy compounds (e.g., vinyl chloride, benzene, lead), the values have decreased over the time due to a better characterization of their toxicity. It is also the case that fewer guidance values have been developed for emerging chemicals of interest (e.g., poly- and perfluoroalkyl substances), where a paucity of available data might not allow a complete characterization of their toxicity and further correlation with certain health effects. Thus, the derived/or to-be-derived guidance values might need further revision over time availability of data increase. When the body of scientific evidence (also the availability of epidemiological, toxicodynamic, and toxicokinetic data) is sufficient to quantify an effect threshold with certainty for a chemical, a new or updated HB2GV can be derived.

## 5. Conclusions

To our knowledge, the HB2GV Dashboard is the first open-access, curated database of human biomonitoring guidance values developed for use in interpreting human biomonitoring data. It allows user-friendly searches for specific chemicals and/or biomarkers of exposure. It also allows users to compare biomonitoring data to the Dashboard values and provides standardized outputs in the form of figures and descriptive text. This new resource can assist global HBM data users such as risk assessors, risk managers and biomonitoring programs with a readily available compilation of guidance values. An additional value of the Dashboard is to facilitate future HBM efforts by helping to identify knowledge gaps and areas where more research could be conducted to improve reliance of GV estimates.

The creation of the Dashboard is a key tangible achievement of the i-HBM Working Group. It is our hope that the Dashboard will become a vital tool for the international harmonization of HBM data interpretation and will help public and governmental agencies better understanding the value of HBM. The i-HBM Working Group also envisages collaborations among international organizations such as the World Health Organization to further promote the use of population level human biomonitoring.

## Figures and Tables

**Table 1:** Human Biomonitoring Health-based Guidance Value (HB2GV) terminology.

Type of Guidance Value (GV)	Definition
Health GV	Derived from epidemiological studies establishing a quantitative relationship between biomonitoring levels in humans and an observed biological response. Exceedance at the individual or population level indicates a need for medical follow-up, and for jurisdictions to identify and mitigate sources of exposure.^a^
Biomonitoring Equivalent (BE)	Concentration or range of concentrations of a chemical or its metabolites that is consistent with an existing value such as a reference dose (RfD) or tolerable daily intake (TDI) for non-cancer endpoints and risk-specific doses for cancer endpoints (Hays et al., 2008). BEs are meant to be used as a screening value to inform chemical prioritization for risk assessment or risk management, and they cannot be used to evaluate the likelihood of adverse health effects or for diagnostic purposes (LaKind et al., 2008). When a human biomonitoring value is above the BE value, it may inform chemical prioritization for follow up, along with investigating underlying exposure sources (Aylward et al., 2013; St-Amand et al., 2014; Faure et al., 2020).
Human Biomonitoring (HBM-I and HBM-II) Values	Values are health-related guidance values derived by the German Human Biomonitoring Commission (Apel et al., 2017; Schulz et al., 2012). They may be derived from epidemiological studies, or similar to BEs, by toxicokinetic extrapolation of tolerable intakes, such as acceptable daily intakes (ADI), TDI, or by toxicokinetic extrapolation of TDI-like values derived from animal experiments. The HBM-I value represents the concentration below which there is no risk for adverse health effects and, consequently, no need for action. For a chemical with a concentration higher than the HBM-I and lower than the HBM-II value, the potential sources of exposure should be identified and either eliminated or reduced by appropriate means. The HBM-II value represents the concentration above which, according to the knowledge and judgement of the commission, there is an increased risk for adverse health effects and, consequently, an urgent need to reduce exposure and to provide individual biomedical care.
Human Biomonitoring Guidance Value (HBM-GV)	In reference to the general population, the HBM-GV refers to the concentration of a substance or its metabolites in human biological material at and below which there is no risk of health effects anticipated for a lifetime exposure (Apel et al., 2020). Any population exceeding an HBM-GV may be prioritized for risk management follow-up. These HBM-GVs are equivalent to the HBM-I values and BE values.

a The Centers for Disease Control and Prevention has set a blood lead “reference value” of 3.5 μg/dL, equivalent to the 97.5th percentile of the blood lead values among U.S. children aged 1–5 years from recent NHANES surveys (Centers for Disease Control and Prevention, 2021). This value describes a cut point for children who have high levels of blood lead compared to a nationally representative group of children but does not provide information on what this level might indicate for children’s health.

## Abbreviations

ADI: Acceptable Daily Intake
BE: Biomonitoring Equivalent
CHMS: Canadian Health Measures Survey
DEHP: di-(2-ethylhexyl) phthalate
GV: Guidance Value
HB2GV: Human Biomonitoring Health-Based Guidance Value
HBM: Human Biomonitoring
HBM-GV: Human Biomonitoring Guidance Value
HBM4EU: Human Biomonitoring for Europe
i-HBM: International Human Biomonitoring Working Group
IBN: International Biomonitoring Network
ISES: International Society of Exposure Science
LOD: Limit of Detection
LOQ: Limit of Quantitation
NHANES: National Health and Nutrition Examination Survey
PBPK: Physiologically-Based Pharmacokinetic
PFHxS: Perfluorohexane sulfonate
RfD: Reference Dose
RV: Reference Value
QA/QC: Quality Assurance and Quality Control
TDI: Tolerable Daily Intake
